# A qualitative study exploring stakeholders’ perceptions of registry-based randomised controlled trials capacity and capability in Australia

**DOI:** 10.1186/s13063-024-08668-8

**Published:** 2024-12-18

**Authors:** Bill Karanatsios, Khic-Houy Prang, Justin M. Yeung, Peter Gibbs

**Affiliations:** 1 Department of Surgery, The University of Melbourne, Parkville, VIC Australia; 2 Western Health Chronic Disease Alliance, Western Health, St Albans, VIC, Australia; 3 Centre for Health Policy, Melbourne School of Population and Global Health, The University of Melbourne, Parkville, VIC Australia; 4 Department of Colorectal Surgery, Footscray Hospital, Western Health, Footscray, VIC Australia; 5 Personalised Oncology Division, The Walter and Eliza Hall Institute of Medical Research, Parkville, VIC Australia; 6 Department of Medical Biology, The University of Melbourne, Parkville, VIC Australia; 7 Department of Medical Oncology, Western Health, Sunshine, VIC Australia

**Keywords:** Registry-based randomised controlled trials, Registry, Qualitative study

## Abstract

**Background:**

Traditional randomised controlled trials (RCTs) are the gold standard for evaluating the effectiveness of interventions in clinical research. Traditional RCTs however are complex, expensive and have low external validity. Registry-based randomised controlled trials (RRCTs) are an emerging alternative approach that integrates the internal validity of a traditional RCT with the external validity of a clinical registry by recruiting more real-world patients and leveraging an existing registry platform for data collection. As RRCTs are a novel research design, there is limited understanding of the RRCT landscape in Australia. This qualitative study aims to explore the RRCT landscape in Australia including current capacity and capabilities, and to identify challenges and opportunities for conducting RRCTs.

**Methods:**

We conducted 30 semi-structured interviews with 18 clinician researchers, 6 research program managers and 6 research governance officers. Interviews were audio-recorded and transcribed verbatim. We analysed the data using thematic analysis.

**Results:**

We identified four overarching themes: (1) understanding of the RRCT methodology concept and knowledge of Australian clinical registries and RRCT landscape; (2) enablers and barriers in the uptake and conduct of RRCTs; (3) ethics and governance requirements impacting the conduct of RRCTs and (4) recommendations for the promotion, support and implementation of RRCTs. Understanding of and ability to define an RRCT varied considerably amongst participants, as did their appreciation of the role the registry should play in supporting these trials. Lack of ongoing funding to support both registries and RRCTs, along with low awareness and minimal education around this methodology, were identified as the predominant barriers to the uptake of RRCTs in Australia. The simplicity of RRCTs, specifically their pragmatic nature and lower costs, was identified as one of their best attributes. There was consensus that inadequate funding, onerous research governance requirements and poor awareness of this methodology were currently prohibitive in enticing clinicians and researchers to conduct RRCTs. Recommendations to improve the uptake of RRCTs included establishing a sustainable funding model for both registries and RRCTs, harmonising governance requirements across jurisdictions and increasing awareness of RRCTs through education initiatives.

**Conclusions:**

RRCTs in Australia are an evolving methodology with slow but steady uptake across a number of clinical disciplines. Whilst RRCTs are increasingly identified as a beneficial alternative methodology to evaluate and improve current standards of care, several barriers to effective RRCT implementation were identified. Creating greater awareness of the benefits of RRCTs across a number of stakeholders to help secure ongoing funding and addressing both registry and RRCT governance challenges are two essential steps in enhancing the uptake of RRCTs in Australia and internationally.

**Supplementary Information:**

The online version contains supplementary material available at 10.1186/s13063-024-08668-8.

## Introduction

Traditional randomised controlled trials (RCTs) are considered the gold standard for evaluating the effectiveness of interventions in clinical research [1, 2]. Traditional RCTs are expensive and complex to perform. They enrol a highly selected population through strict inclusion criteria, impacting their generalisability with a high proportion of trials failing to meet recruitment goals [3, 4].

Alternative research methodologies have been developed in pursuit of more affordable and generalisable high-quality clinical evidence [3, 5]. One such alternative methodology is the registry-based randomised controlled trial (RRCT). RRCTs encompass a broad and varied definition whereby data is obtained from or collected into a registry. A registry is defined as ‘an organised system that uses observational study methods to collect uniform data to evaluate specified outcomes for a population defined by a particular disease, condition, or exposure’ [6]. A registry can support an RRCT in various ways, including by facilitating participant identification, participant recruitment, or capture of patient detail and outcome measures in the registry. Participant randomisation can occur either within or outside the registry. RRCTs share a number of common features with traditional RCTs such as patient stratification and randomisation [7], but depending on what role the registry plays, they are usually best suited for testing hypotheses involving approved clinical interventions for which there are uncertainties about the optimal sequence, duration or combination of standard-of-care treatment, or where multiple standard-of-care options exist that have not previously been compared head to head [2].

The potential benefits that RRCTs offer have been well documented across a number of reviews that have been conducted on RRCT implementation [11, 12]. These include ease of recruitment, broad inclusion criteria, large sample size, long-term follow-up, generalisability of findings and cost effectiveness [2, 13]. Previous reviews [2, 13, 14] found that a large number of RRCTs are being conducted in Scandinavian and North American countries where their research infrastructure and well established national registries with strong data linkage capabilities with external data sources are best suited to support such studies.

One Scandinavian study that demonstrated the true potential of RRCTs is the *Thrombus Aspiration during ST-Segment Elevation Myocardial Infarction* (*TASTE*) study [15]. TASTE involved 7400 patients and cost 10% of the budget of an equivalent traditional RCT. Such significant cost savings can be attributed to data collection and operational infrastructure already being in place through existing registries. Further TASTE study cost savings, in comparison to traditional RCTs, are attributed to reduced trial-specific visits, site start-up expenses and trial monitoring requirements [11]. RRCTs have the potential to inform and change clinical practice as demonstrated by the TASTE trial which resulted in a rapid decrease in the use of thrombus aspiration in STEMI patients undergoing percutaneous coronary intervention (PCI) in Sweden [16]. The Validate Swedeheart trial also demonstrated similar findings confirming that the efficacy of certain treatment choices was equal to their more expensive alternative [17].

Despite their advantages, RRCTs have a number of limitations that are predominantly underpinned around the robustness of the registry and the registry data supporting them. The most commonly reported limitations are insufficient or incomplete registry data, non-uniformity of data collection [2, 18] and access to registry data due to privacy issues [18].

In Australia, RRCTs represent less than 1% of the total RCTs registered on the Australian New Zealand Clinical Trials Registry (ANZCTR) and this substantiates the findings by Ahern et al. [14] and Yan et al. [19] that RRCTs are an emerging methodology and predominantly confined to non-commercial investigator or collaborative group led studies. In 2022, Ahern et al. searched the ANZCTR by using the search term ‘registry’ and study type ‘interventional’ and identified 20 trials that used a clinical registry to support the RCT in some capacity [14]. Of these 20 trials, we ascertained that 16 would meet the broader criteria of an RRCT whereby the registry is used for either identifying, recruiting, randomising or collecting trial outcomes. However, if we were to use a stricter definition of an RRCT as per Li et al. [20] whereby eligible patients are identified and recruited from the registry, the patients’ existing baseline medical history is recorded in the registry and data related to the intervention and the outcomes are all captured in the registry, then none of these registry trials would be considered an RRCT. Studies that only used the registry for post trial follow-up or confirmation of disease or treatment status were excluded. We conducted an updated search on ANZCTR in 2024 using the search term and variation of ‘*Registry Randomized Clinical Trials*’ and yielded 52 results. When each trial was further assessed against the broader RRCT criteria above, only 11 trials met the criteria (Table 1). Some RRCT eligible studies that were captured in Ahern et al. [14] were not captured under our search terms and vice versa. There were no ANZCTR trials in our search that fulfilled the Li et al. definition of an RRCT.

**Table 1:** ANZCTR registered RRCTs

Discipline	Study name	Registry use	Sponsor/funding
Orthopaedic	RASKAL ACTRN12621000205831	Study data collected in registry	Philanthropic funds
Orthopaedic	Acronym not provided ACTRN12620001251910	Patients identified from registry	Self funded
Nephrology	SWIFT ACTRN12620001061921; ACTRN12618001976279	Patient feedback captured in registry	Government funded (NHMRC)
Oncology	PROpatient ACTRN12619001126101	Study data collected in registry	Government funded
Neurology-stroke	STELAR ACTRN12619001072101	Study data collected in registry	Charity funding
Oncology-upper GI	ALLTRAC ACTRN12618001480279	Study data collected in registry	Collaborative group
Cardiology	FAN Trial ACTRN12618001124224	Patients identified from registry	Charity funding
Neurology-stroke	Prevent Second Stroke (P2S) ACTRN12617001205325	Patients recruited from registry	Government
Emergency department	RAPID-TnT ACTRN12615001379505	Study data collected in registry	Collaborative groups NHMRC Commercial sponsor support
Urology	No acronym ACTRN12615001369516	PROMS collected in registry	Government funded
Cardiac surgery	PORTICO-IDE Registered on clinicaltrials.gov NCT02000115	Patients identified from registry	Commercially sponsored

Ten RRCTs were non-commercially funded studies, predominantly being funded by the National Health and Medical Research Council (NHMRC) grants or by other government or philanthropic funds. The role of the registry was variable with patient identification, participant recruitment and study data collection being the prevalent use of the registry. Capturing all studies that may fall under the definition of an RRCT presents a number of challenges as not all RRCTs are registered under the banner of an RRCT or easily searchable by using commonly used search definitions for RRCTs. This is further compounded by the fact that not all studies are registered on ANZCTR. As such it is difficult to accurately estimate the number of RRCTs currently being conducted in Australia and more broadly internationally and does highlight the need for a more prescribed definition of what characteristics should attach to an RRCT, particularly around how a registry is employed.

### Objectives of study

As the RRCT is a relatively new clinical trial methodology, the need to gain greater understanding of the RRCT landscape in Australia is important in an effort to identify what is limiting uptake of this methodology. Given that RRCTs are governed by the same regulatory requirements of traditional RCTs which comprises ethical, governance and legal considerations, it is important to get the perspectives of a broad range of stakeholders to understand how this impacts RRCT uptake and implementation. The focus of our study is on stakeholders rather than on end-users acceptance and participation in RRCTs as this has been previously explored [21].

Using a qualitative approach, this study aims to explore the RRCT landscape in Australia including current capacity and capabilities, and to identify challenges and opportunities for conducting RRCTs from the perspectives of clinicians, research program managers and research governance officers. This understanding will inform strategies that help to create greater awareness of RRCTs and to enhance RRCT acceptability and uptake in Australia and internationally.

## Methods

### Study design

This study used a qualitative approach to capture the experience and attitudes of clinicians, research program managers and research governance officers in relation to RRCTs, considering their role at their respective hospital or academic institution. Semi-structured interviews with participants were conducted using an interview guide. The interview guide was designed to elicit an understanding of awareness, feasibility and acceptability of conducting RRCTs in Australia including potential barriers and enablers to effective RRCT implementation. Participants were invited to make additional comments to ensure that all topics they wished to discuss were covered. The qualitative study is reported in line with guidelines set out in consolidated criteria for reporting qualitative research (COREQ) [22] (Appendix 1).

### Recruitment of participants

Maximum variation sampling and snowballing were employed to identify and select eligible participants [23]. This is a type of purposive sampling technique that aims to explore a wide range of perspectives. All participants were eligible for interview if they were involved in the conduct or support of clinical trials within a healthcare setting in Australia and internationally without the prerequisite of any current or prior direct experience with RRCTs. However, prior experience or association with supporting or administering clinical trials or a registry was considered essential to ensure that perspectives could be meaningful and informative. The eligible participants were defined by the nature of their association in supporting clinical trials or a registry at their respective institution as either a clinician researcher, research governance officer, registry/data custodian or research program manager. Hereafter, the latter two profession classifications are broadly referred to as ‘*research administrators*’. Australian participants were recruited from five major Melbourne metropolitan hospitals and two regional hospitals some of which were identified through their publications on registry-based trials in Australia and through existing networks of this paper’s co-authors. Identified eligible participants were then invited via email to be interviewed. During the interview, participants were asked if they can recommend other key experts in this space. International participants were identified through their publication output on RRCTs and were included to get an international perspective against the study aims and to ascertain degree of alignment with Australian perspectives. A total of 32 participants from Australia and 4 international participants were invited to participate via email, of which six did not respond despite follow-up invites.

### Data collection

Thirty interviews were undertaken between October 2020 and March 2023 by one researcher (BK) (BAppSc Hons in Medical Laboratory Science) who is a health research ethics and governance administrator undertaking their PhD investigating the Australian RRCT and registry landscape, including identifying the barriers and enablers for the uptake and implementation of RRCTs. A semi-structured interview guide was used that was informed from two prior scoping reviews [2, 24]. The interview guide was further refined after the first couple of interviews. After 23 interviews, no more new information was being presented and it was therefore agreed that data saturation had been reached and we would complete the remaining seven interviews that were already scheduled. Due to COVID-19 related social distancing requirements, most of the participants were interviewed via zoom with only eight interviews conducted face to face at a venue of their choice. Interviews averaged 35 min (range 15 to 85 min) in length. All interviews were audio-recorded with the participant’s consent. Interview recordings were transcribed verbatim by a professional transcription service and imported into QSR NVivo 12 for coding and storing.

### Data analysis

Participants were grouped into five categories post interview by the interviewer as either RRCT practitioner, RRCT proficient, RRCT aware, RRCT beginner or RRCT novice based on their understanding and/or experience with the RRCT methodology concept, how they defined an RRCT and how a registry is able to support an RRCT and their involvement with RRCTs (Table 2). This categorisation across all participant group helps capture their level of understanding, awareness and overall competency of this methodology and helps identify any variability across and within the participant groups.

**Table 2:** RRCT competency categories

Category	Definition
RRCT practitioner	Participants who were directly involved in the conduct of an RRCT or who currently or previously supported an RRCT through their role of employment and who could clearly articulate the concept and the various ways the registry could support an RRCT. They could name RRCTs that they were involved with
RRCT proficient	Participants who may have supported an RRCT through their role of employment and who articulated the various ways a registry could support an RRCT and could name a registry that has supported an RRCT
RRCT aware	Participants who were familiar with the concept and were able to articulate some of the aspects of how a registry can support an RRCT. No prior involvement with RRCTs
RRCT beginner	Participants who had some idea of what an RRCT was but were unable to clearly articulate the various ways a registry could be used to support an RRCT. They could not name any RRCTs
RRCT novice	Participants who had no awareness of RRCT methodology

Themes were identified, analysed and reported within the data and across participant groups, using a combination of inductive and deductive coding as part of our thematic analysis [25, 26]. Two researchers (BK and KP) independently analysed five interview transcripts using a coding tree developed from the structure of the interview questions. The researchers’ resulting coding trees were compared and through further discussion between the researchers amended accordingly until consensus on an agreed coding tree was reached. The remaining interview transcripts were then coded by one researcher (BK) based on the agreed coding tree, and emergent themes were discussed between the authors and added as needed. Codes that were similar were clustered together and subsequently collapsed into emergent themes as part of the theme development and revision process.

*Ethical considerations.*

Ethical approval for this study was granted by the University of Melbourne, Human Research Ethics Committee ( ID 1954874). Written consent was obtained from all participants prior to data collection to record and use their interview data.

## Results

### Participants

Thirty (83%) of the 36 participants invited were interviewed. Tables 3 and 4 show the demographic characteristics of the participants. This included 18 clinicians across several specialties (two of which were also registry custodians and one part-time chief informatics officer); six research program managers (two of which manage national data platforms); and six research governance officers within the Office for Research in a public health service overseeing ethics and governance of research at their respective institutions (one also administered registries). Participants exhibited varied levels of understanding around the RRCT methodology with clinicians showing the greatest RRCT awareness and research governance officers the least.

**Table 3:** Participant professional classification and demographics

	Clinicians (***n*** = 18)	Research governance officers (***n*** = 6)	Research administrators (***n*** = 6)
**Gender**			
Male	12	2	4
Female	6	4	2
**Age groups**			
25–34			
35–44	8	3	2
45–54	8	2	4
55–64	1		
65 +	1	1	
**Sub-speciality/role**			
Medical oncology	5		
Surgeons	3		
Others^a^	10		
Research governance officer (RGO)		6	
Research data custodian/program manager (RPM)			6

^a^Epidemiology, cardiology, neurology, intensive care, primary care, nephrology, health administration and paediatrics

**Table 4:** Participant RRCT competency

	RRCT competency				
	Practitioner	Proficient	Aware	Beginner	Novice
**Medical oncology**	5				
**Surgeons**			3		
**Others**^**a**^	5	2	3		
**Research governance officer (RGO)**	1		2	2	1
**Research data custodian/program manager (RPM)**	2	1	2	1	

^a^Epidemiology, cardiology, neurology, intensive care, primary care, nephrology, health administration and paediatrics

### Themes

Four overarching themes were identified from participant interview responses regarding their knowledge and awareness of (1) the current registry and RRCT landscape in Australia; (2) enablers and barriers in the conduct of RRCTs; (3) ethical and governance issues impacting the conduct of RRCTs and (4) recommendations for the promotion, support and implementation of RRCTs in the future. The overarching themes and sub-themes by registry and RRCT are presented separately in Table 5.

**Table 5:** Overarching themes and sub-themes

Overarching themes	Sub-themes			
	**Registry**		**RRCT**	
Australian clinical registries and RRCT landscape	Registry funding	• Registries are not well supported or funded• Registry data quality dependent on funding	RRCT awareness	• Variable awareness of RRCTs being conducted• Awareness of RRCTs confined to within area of expertise• Greatest awareness held by trialists and registry custodians
	Registry type	• Most registries are clinical registries	RRCT definition	• RRCT definition provided was broad and varied • Consensus not to restrict RRCTs to a single definition • RRCTs can be supported by a registry in many different ways
	Electronic medical records (EMR)	• EMR not suited to replace registries in near future		
Enablers	Registry supporting RRCT	• Patient identification and recruitment • Collection of baseline and outcome data • Facilitate long-term follow-up	RRCT benefits	• More efficient, simple and cost effective • Greater external validity of trial outcomes • Help answer questions of public health interest
Barriers	Registry cost/funding	• Registries expensive to establish and maintain • Registries not adequately resourced or financed • Predominance of clinical registries	RRCT funding	• Not adequately funded • Competing for participants by well-funded commercially sponsored RCTs
	Registry limitations	• Registry data quality • Lack of value proposition for all contributing to the registry • Do not capture SAEs • Not suitable end points collected	RRCT awareness	• Unknown methodology
	Registry environment	• Registry steering committees not having a research focus • Federated system	RRCT scope	• Not suited for novel/early phase clinical studies
Ethics and governance of registries and RRCTs	Registry consent	• Opt out consent acceptable	RRCT consent	• Participant consent required due to randomisation
	Registry governance	• Cross jurisdictional registry governance requirements challenging—including access to registry data	RRCT governance	• Research governance bureaucracy prohibitive • Burdensome research governance requirements for level of funding and trial risk profile
	Registry ethics	• Current ethical review arrangements not a concern	RRCT ethical review	• Current ethics requirements deemed appropriate • Opportunity however to explore lesser review pathways based on trial risk profile
Recommendations	Education	• Educate registry steering committees to facilitate better access to registries for research purposes	Education/awareness	• RRCT education and training to increase methodology understanding and awareness
	Government support	• Government to prioritise funding and support for registries • Government to guide a registry funding model involving all potential registry users/stakeholders	Government support	• Government to prioritise funding and support for RRCTs • Streamline ethical review of RRCTs based on risk profile • Reduce governance administrative burden • Harmonise jurisdictional research governance requirement • RRCT activity as quality indicator for health services
			Institutional support	• Embed RRCTs at an organisational level

#### Theme 1: understanding of the RRCT methodology and concept, and knowledge of the Australian clinical registry and RRCT landscape

##### RRCT definition

The definition of an RRCT varied amongst all participants interviewed and was predominantly underpinned around the role of the registry in supporting the RRCT. *Practitioner* or *proficient* level RRCT participants were more likely to define an RRCT as a trial that is supported by a robust registry that facilitates patient identification, recruitment or a collection of basic clinical and trial outcome data or a combination thereof. Randomisation could occur within or outside the registry.

*I would describe them [RRCT] as, innovative, and pragmatic way of ensuring data from each patient is used wisely. I would say that to me, the gold standard for a registry-based trial would be a very clear, comprehensive, prospective registry that’s already been built, already has the IT system, already has key players in the hospital system, involved and interested. And then from there to build a trial- a randomized trial into the registry allowing patients on the trial to be compared to patients that are already existing on the registry.* (Clinician 1).

*Aware* and *beginner* practitioners provided a broader definition of what an RRCT could be and were more likely to limit the role of the registry to either one of only identifying patients, recruiting patients or collecting an outcome(s) in the registry. Some participants declared complete ignorance. Overall, there was consensus that a registry can support an RRCT in many different ways and applying a strict definition could be counterproductive, and could potentially ‘cause trouble’ because it would limit the scope of an RRCT.

##### RRCT awareness

Awareness of what RRCTs were being conducted was variable amongst participants, with greater awareness held for trials that were conducted within their discipline of practice or where there was a close association with an institute or colleagues who were actively involved in conducting RRCTs. Even RRCT practitioners were relatively unfamiliar with Australian RRCTs outside of their own clinical discipline. Research governance officers (RGO) had the ‘least awareness’ of RRCTs and were less likely to name an RRCT given their indirect involvement in supporting RRCTs. Research program managers exhibited varied awareness and was dependant on if they supported registries or data used to conduct RRCTs. Clinicians that had an affiliation with a state or national registry were more likely to declare a greater level of RRCT awareness. Some participants across all stakeholder categories stated they had awareness of RRCTs being conducted but could not name a specific RRCT.

*I definitely think a larger awareness campaign needs to be done because before you got in touch about them (RRCTs), I didn’t really know.* (RGO 1).

##### Australian registry landscape

There was general consensus amongst the participants that clinical registries in Australia overall are not well organised and there are only a limited number of disease or procedure specific registries that are well funded and supported. These registries tend to be national registries that have been in place for many years. Most participants acknowledged that registry data quality was associated with the amount of funding and resourcing made available to a registry. Australia’s federated system with its cross-jurisdictional regulatory variability was attributed by some as a significant reason why Australian registries are generally not well organised and supported. It was also noted that this is further compounded by the public–private health system divide and the overall data flow disconnect with primary care providers.

*I don’t think that we’re as mature as some of those (Scandinavian) countries. We’ve certainly got a way to go. There are some great registries, but not many, and I think it’s still difficult to undertake for a lot of areas.* (RGO 2).

Whereby it was acknowledged that electronic medical records (EMR) could play a pivotal role in supporting RRCTs, the current state of play and variability in how EMRs are configured, implemented, supported and utilised across health services makes this a distant proposition.

*You’d have to set up all of the process so that it is systematic, and you’d have to really train and do a major cultural change. I think it’s possible, but I think it would have to happen over many, many, many years to get there.* (RGO 3).

#### Theme 2: enablers and barriers in the uptake and conduct of RRCTs

Five enablers and six barriers were identified. RRCTs present a number of benefits predominantly around being simple and efficient to conduct, have reduced trial related costs, provide generalisability of findings which is somewhat lacking in traditional RCTs and help answer questions of public health interest. However, RRCTs present a number of barriers which are associated with inadequate funding and support for registries and RRCTs, recruitment challenges against traditional RCTs, lack of awareness of methodology, not being suitable for early phase trials and burdensome governance processes.

##### Enablers

The majority of participants appreciated that RRCTs provide a number of advantages over traditional RCTs. Most participants see the simplicity, efficiency and cost effectiveness of RRCTs over traditional RCTs as one of their greatest attractions. They appreciated that RRCTs usually compare two standards of care, greatly enhance the generalisability of the trial findings and offer public interest benefit unlike traditional RCTs as they are not underpinned around commercial imperatives. They acknowledged that well established registries should be able to accommodate patient identification, recruitment and collection of baseline and trial outcome data therefore negating further data collection forms and administrative workload. Participants also noted that the registry could provide more accurate participant numbers to assist with performing feasibility estimates.

*You should run everything within the registry because the whole point of the registry is that you don’t have to do anything extra, you can just simply use the patient’s existing [data] collection within the registry to answer standard of care questions that would otherwise take a lot of effort to set up outside the registry.* (Clinician 2).

##### Barriers

The majority of participants identified the lack of financial support to accommodate staffing and other resources for both registries and RRCTs as one of the biggest barriers in the conduct of RRCTs. Although RRCTs are considered to be more efficient to conduct than traditional RCTs, this was prefaced around an already existing and well supported registry being in place to support the RRCT. Furthermore, as most Australian registries are clinical quality registries that support quality and safety initiatives, it was recognised that the data points they collect may not always be suitable to support an RRCT. Therefore, the funding and resources required to establish and maintain a suitable high quality registry was recognised as one of the biggest challenges for RRCTs. The lack of substantive commercial sponsorship or a clear government funding agenda for registries and RRCTs more broadly was identified as contributing significantly to this.

*Even when I think about the ones (registries) that we have at (a Melbourne hospital), the quality is variable depending on how much funding they’ve had and therefore what resources they’ve had. We’ve got a (rare disease) register, which is the biggest in Australia…. Even then, it’s so under-resourced that their data quality is not as good as it could be, even though it’s seen as such a valuable resource.* (RGO 2).

Lack of awareness and education around this methodology was identified by many participants as a limiting factor to the uptake of RRCTs. This was further compounded by the perception that an institution contributing to a multisite registry may not necessarily receive any direct benefit such as being part of a registry trial or being informed early on of RRCT outputs to help guide local clinical practice.

It was well acknowledged that registries play a critical role in supporting an RRCT and can directly impact their uptake and how RRCTs are perceived. Given that the majority of registries are not built to capture and report on serious adverse events, it was acknowledged by most that RRCTs are not well suited to test novel therapies in early phases of clinical trial studies. This was identified as a potential deterrent for some clinicians who may only be interested in working with new drugs or devices. It was noted that this may influence clinicians in giving preference to a commercially sponsored RCT if an RRCT was also on offer therefore impacting RRCT recruitment capability.

*I think that registries would be more appropriate for interventions that already have some reasonable phase one phase two data. […..] I think the amount of oversight you need to do for these first-in-man studies is probably over and above what a registry would provide.* (Clinician 3).

The above limitations have the potential to relegate RRCTs predominantly to regional centres where there are fewer commercially sponsored RCTs being offered or confine RRCTs to a select group of clinicians who appreciate what they can offer.

#### Theme 3: ethical and governance requirements impacting the conduct of RRCTs

Registry governance requirements associated with establishing a registry and collecting data, or seeking permission for access to registry data was identified as a significant barrier to the conduct of RRCTs. Opt out consent for participation in a registry was considered appropriate by most participants and this is consistent with current practice of participant enrolment in a registry.

Registry and RRCT governance requirements were identified as being highly bureaucratic potentially discouraging clinicians from conducting RRCTs. Many participants felt that the governance burden associated with conducting RRCTs was disproportionate to the level of risk they presented. Some felt that the lack of national harmonisation of the governance process for both registries and RRCTs further compounded the situation. Whilst there was collective agreement around the burden imposed by governance bureaucracy, the issue of ethics review of RRCTs provided a mixed response. A number of participants felt that current RRCT ethics review requirements were appropriate given that the methodology randomised patients across two intervention arms. Others felt that given that RRCTs were predominantly used to compare two standards of care, the risk profile of RRCTs could justify their review under a less rigorous review pathway such as the Low Risk Ethics Panel (LREP) process. Responses around participant consent did not change much even when a distinction between randomising participants or cluster randomisation was discussed. There was general consensus amongst those interviewed that some form of consent to participate in the RRCT was required due to the introduction of randomisation, as randomisation denied the participant selection choice between available treatment options. Despite the evolving international debate on the role of consent for pragmatic trials and the need to review current consenting requirements when the risk for participants is considered to be low [27], in Australia ethics committees currently rarely delineate between traditional and pragmatic RCTs in this regard. Based on some responses from interviewers and increasing articles calling for international consensus and reform around this space [27–31], consent for pragmatic trials is an evolving matter and hopefully one that lands on a pragmatic consenting solution which is risk assessment based. Overall current ethics requirements were not identified as onerous or overly prohibitive but responses did highlight the opportunity to pursue a more simplified review pathway commensurate to the RRCT risk profile.

*So the question I would have is, is that truly an intervention? I would argue it’s not, and if it’s not, then I don’t think it would have to go through high-risk ethics. That would be my feeling about them.* (Clinician 4).

*In terms of ethics though, it’s a little bit more complex. If there is a pure pragmatic-comparative effectiveness study where you’re comparing two things which would be done, used anyway, then I think the ethical considerations probably aren’t that great. In fact, I think there’d be a good case that you could make to an ethics committee to give you a waiver of consent, or at least delayed consent.* (Clinician 5).

#### Theme 4: recommendations for the promotion, support and implementation of RRCTs

Participants acknowledged that RRCTs both globally and in Australia are an emerging methodology and provided the following suggestions that could help promote and increase the uptake of RRCTs in Australia: education and awareness, broad stakeholder engagement, government funding support and advocacy.

Raising awareness and providing education opportunities would help advance the profile of RRCTs. This could be achieved in part by conducting and publishing further landmark studies and also leveraging off the validation provided by prior landmark studies like TASTE [15].

As it is important to demonstrate that RRCTs can be impactful, an integral part of the education process would be to emphasise the importance that the research question and design of the RRCT must be simple and that the outcomes collected in the registry will help answer the research question and provide a clear health outcome. RRCTs that are too ambitious, complex or are not designed with due consideration to the registry limitations are set up to fail unless other workarounds are put in place. Education would be integral in ensuring that this research methodology is utilised in the best possible way in order to be impactful and to continue providing the methodology validation. It was also proposed that education would need to extend to registry steering committees which currently do not have a ‘research focus’ and may therefore make accessing registry data for research purposes more difficult.

*I guess that’s something I’ve come to understand more as these sort of opportunities are opening up to us, is that the existing registry steering committees you know, don’t necessarily have that (research) focus.* (Clinician 6).

Participants broadly acknowledged that RRCTs involve many and varied stakeholders that needed to be actively engaged. Ultimately RRCTs need to not only be embedded in the culture of a department but the institution as a whole and ultimately more broadly across the healthcare system. A national approach would be the ultimate goal. This will help address certain challenges around registry data quality, access to registry data, governance processes and consenting of patients into an RRCT.

*The most important aspect of an embedded trial is it’s one thing to embed it into the record, but you need to then embed it into the culture of the department. If you’re going to rely on the clinician to consent, then all the clinicians have to agree that it’s a worthwhile thing to do. It has to be embedded into the workflow, has to be embedded into the psyche of the clinicians, and it has to be embedded in the record. The embedding in the record is the easiest.* (Clinician 7).

Participants proposed that governments can assist with this by providing infrastructure support and funding of registries and RRCTs as a healthcare priority. It was acknowledged that in parts this was now been fulfilled through the Medical Research Future Fund (MRFF) offering grant opportunities to support RRCTs [32]. Most participants noted that there is a role for government in helping develop a registry funding model that could seek contribution from the various public and private sector stakeholders toward sustainable registries that are well suited to support RRCTs.

*I think the only sustainable way forward is for government to actually fund each tumor stream to have a registry.* (Clinician 2).

## Discussion

In this qualitative study which explores awareness, perceptions and understanding of the RRCT landscape in Australia, we found that inadequate funding to support registries and RRCTs, poor awareness and education around the RRCT methodology, and having to compete against well funded traditional RCTs for patients are some of the major barriers in the conduct of RRCTs.

Our findings from the interviews conducted identified similar themes around the enablers and barriers to the conduct of RRCTs as were identified in our two earlier scoping reviews we conducted that also explored the global RRCT landscape [2, 24], and also aligned with findings from expert interviews conducted by the Clinical Trials Transformation Initiative on registry trials [33]. The quality of the registry data, funding for the ongoing maintenance of registries, simplified regulatory requirements and governmental or other appropriate support for this type of trials are ubiquitous needs across all jurisdictions.

Our findings around RRCT recruitment challenges and barriers aligned with the findings from a qualitative study [21] exploring the feasibility and acceptability of RRCTs amongst cancer patients and clinicians. As patients are an integral stakeholder in RRCTs and can strongly influence trial recruitment potential, the involvement of patients or suitable consumers early on in the development of the RRCT protocol could assist with recruiting patients into the trial and help create greater public awareness. Consumer engagement in the design and implementation of trials in Australia is now a requirement under the Australian Commission on Safety and Quality in Health Care, The National Clinical Trials Governance Framework [34].

We identified that RRCT awareness and activity was widely variable and dependent on the institution the participants belonged to and their respective role. Clinicians from institutions with high clinical trial activities were more likely to define what an RRCT is correctly and to be aware of at least one RRCT being conducted in Australia. Research governance officers and to a lesser extent research program managers were less likely to be able to define what an RRCT is and exhibited the least amount of RRCT awareness. They were more likely to provide non-specific responses to the questions asked, such as being able to name an RRCT or a registry that was used to support it.

There was greater alignment amongst the participants in relation to RRCT barriers and enablers and potential strategies to address them. Promotion of RRCTs through education materials and workshops will help to inform and demonstrate the benefits of this methodology. The participants were not explicitly asked as to which entity should assume the role of education provider. However, entities that are well advanced in the conduct of RRCTs are possibly best placed to facilitate education and training around this methodology. In Australia, the Victorian Comprehensive Cancer Centre (VCCC) Alliance-Centre for Cancer Education has developed education materials on registry-based trials [35]. Similarly, the Australian Clinical Trials Alliance (ACTA) has been promoting education workshops and forums around registry-based trials [36].

There was overall consensus that RRCTs are a valuable methodology in need of greater promotion and support as they help answer simple but important public health questions. This is underpinned around RRCTs recruiting real-world patients and having the benefit of integrating the internal validity of a traditional RCT with the external validity of a clinical registry. This is consistent with findings from a number of publications promoting the ‘real world’ benefits of pragmatic trials, which are underpinned on the enhanced generalisability of their outcomes [5, 37].

Inadequate funding and resources to support registries and RRCTs was one of the biggest barriers identified as impacting their uptake. As RRCTs are largely investigator initiated studies and are predominantly funded by non-commercial means, the role of government in establishing a funding model to ensure the sustainability of registries and RRCTs is important. As commercially sponsored RCTs are adequately funded, the lack of sustainable funding for registries and RRCTs can create reluctance by clinicians to commit to this new methodology. RRCTs registered on the ANZCTR (Table 1) confirm that ultimately RRCTs are initiated and conducted by clinicians and trialists who are truly committed to RRCTs and who are willing to expend the time and energy to pursue highly competitive grants, provide their own funds or pursue other non-commercial funding options to support them. This in turn creates the risk whereby RRCTs may be confined to a smaller number of trialists, therefore maintaining the status quo in relation to their uptake and accessibility. It was beyond the scope of the interviews to identify suitable funding models but this should be an area for further exploration. Government prioritising support for registries and RRCTs as part of the healthcare agenda would be a requisite step in facilitating their greater uptake. The Australian Government’s announcement of the *Medical Research Future Fund (MRFF) 2023 Innovative Trials Guidelines* which will provide $23.7 million Australian dollars toward innovative trials including RRCTs is a step in the right direction [32]. This should provide the support needed to initiate a number of important and potentially landmark RRCTs that may further validate this methodology in Australia.

All RRCTs in Australia must be ethically and scientifically reviewed and approved by a Human Research Ethics Committee (HREC) under the National Statement on Ethical Conduct in Human Research [8]. They also need to fulfil institutional site specific governance requirements to be granted authorisation to be conducted at that institution [9]. Governance requirements predominantly involve research agreements between institutions, study related budgets and approvals from various levels of management. It is a requirement that the registry to support the RRCT has the appropriate ethics approvals and that permission to access the data for research purposes is in accordance with the registry ethics approval and the registry data access governance requirements [10].

There were some concerns expressed with current ethical review requirements for RRCTs. As RRCTs often compare two accepted standards of care, they could be considered for review by a Low Risk Ethics Panel (LREP) rather than High Risk Ethics Panel, but consent for participation remains. This risk benefit approach is also consistent with what is being proposed by Anderson et al. [28], particularly where standard of care and already approved interventions are trialled. Califf and Sugarman argued for the need to re-examine the regulatory and ethical landscape in which pragmatic trials are conducted in order to help facilitate their uptake [4]. In Australia, the single ethical review process streamlines ethics review of trials and to some extent ameliorates potential cross jurisdictional discrepancies as single ethics review by any accredited HREC can be accepted across jurisdictions. Allocation of low risk RRCTs for review by LREPs will help expedite review of trials by institutions that only have a LREP and no HREC but may be best suited for single site studies at those sites as not all LREPs are adequately constituted to facilitate ethical review for multisite studies in the manner accredited HRECs currently do. This is an area that requires further exploration under the auspices of the NHMRC.

Although ethical review of RRCTs presents no major concern, a significant challenge pertains to governance authorisation and the bureaucracy associated with accessing registries, and the institutional governance authorisation requirements for approving the conduct of RRCTs at individual sites. The Australian research landscape is somewhat hindered by the federation system under which certain aspects of research are governed by either federal or state legislation creating governance process variability across jurisdictions resulting in increased bureaucracy. Yan et al. found that jurisdictional boundaries place limitations on RRCTs around data sharing and create the need for multiple approvals [19]. It is anticipated that similar governmental federated systems in other countries like the USA, Canada and Europe would pose similar challenges.

The manner in which EMRs have been implemented across Australia is another prime example of the variability of a federated system presents cross jurisdictions. Where EMRs could potentially one day in part assume the role of a registry, this is inhibited to some extent by the variable EMR systems across the public health care sector. The role of federal government in establishing a funding model for registries and RRCTs, and legislation/policies that provide provisions for addressing the governance challenges that arise from current arrangements would be highly welcomed and would help reduce the administrative burden for clinicians and trialists wanting to access registry data and initiate a multisite RRCT. The benefits gained from the streamlined single ethical review process are somewhat eroded by the persisting and mounting research governance challenges that need urgent attention, but should be used as an example of what can be achieved through good will, appropriate legislation and cooperation across all levels of government.

RRCTs in Australia are progressively being utilised across a number of clinical disciplines as a beneficial alternative methodology to evaluate and improve current standards of care. Despite this increase in their uptake, several barriers to effective RRCT implementation still exist. Better promotion to create greater awareness of the benefits of RRCTs across a number of stakeholders to help secure more ongoing funding, along with addressing both registry and RRCT governance challenges, are some of the essential steps that could increase the uptake of RRCTs in Australia and more broadly.

### Limitations

One of the limitations of this study was the potential for selection bias as those interviewed were participants who were known to the author to be active in the clinical trial and registry environment. As we employed maximum variation sampling and snowballing to identify and select eligible participants, we ran the risk of not including an adequate number of participants who were RRCT naïve. We were only able to interview one participant from a rural/regional setting and therefore findings from our metropolitan participants may not be representative of what is occurring in the regional centres. A follow-up survey to a large and diverse clinical trial active workforce should provide good representativeness in this regard and help determine RRCT awareness and understanding across a number of cohorts and locations. We did not interview a number of other stakeholders that help support RRCTs, such as study coordinators and pharmacists.

## Conclusions

RRCTs are an emerging methodology in Australia as awareness of the benefits they provide is progressively appreciated by clinicians and other stakeholders. Increasing awareness and providing education on this methodology are essential elements in ensuring that RRCTs capture the interest of clinicians and trialists and are therefore a consideration as the alternative methodology to the traditional RCT. Government support and prioritisation of establishing a sustainable funding model for both registries and RRCTs would greatly bridge the current gap.

## Supplementary Information

Supplementary Material 1.

## Data Availability

The datasets generated and analysed during the current study are not publicly available due to participants’ confidentiality.

## Abbreviations

ACTA: Australian Clinical Trials Alliance
ANZCTR: Australian New Zealand Clinical Trials Registry
EMR: Electronic medical record
HREC: Human Research Ethics Committee
LREP: Low Risk Ethics Panel
MRFF: Medical Research Future Fund
NHMRC: National Health and Medical Research Council
PCI: Percutaneous coronary intervention
RPM: Research program manager
RCT: Randomised controlled trial
RGO: Research governance officer
RRCT: Registry-based randomised controlled trial
STEMI: ST-Elevation Myocardial Infarction
TASTE: Thrombus Aspiration in ST-Elevation
VCCC: Victorian Comprehensive Cancer Centre

## References

[1] Tang M, Schaffer A, Pearson SA. Embracing the full spectrum of real-world data for cancer medicines research in Australia. Asia Pac J Clin Oncol. 2019;15(3):186–7. 30747483 10.1111/ajco.13121

[2] Karanatsios B, Prang KH, Verbunt E, Yeung JM, Kelaher M, Gibbs P. Defining key design elements of registry-based randomised controlled trials: a scoping review. Trials. 2020;21(1):552. 32571382 10.1186/s13063-020-04459-z PMC7310018

[3] Bergqvist D, Björck M, Säwe J, Troëng T. Randomized trials or population-based registries. Eur J Vasc Endovasc Surg. 2007;34(3):253–6. 17689818 10.1016/j.ejvs.2007.06.014

[4] Califf RM, Sugarman J. Exploring the ethical and regulatory issues in pragmatic clinical trials. Clin Trials. 2015;12(5):436–41. 26374676 10.1177/1740774515598334 PMC4891194

[5] Barnish MS, Turner S. The value of pragmatic and observational studies in health care and public health. Pragmat Obs Res. 2017;8:49–55. 28546780 10.2147/POR.S137701 PMC5438073

[6] Aljurf M, Rizzo JD, Mohty M, Hussain F, Madrigal A, Pasquini MC, et al. Challenges and opportunities for HSCT outcome registries: perspective from international HSCT registries experts. Bone Marrow Transplant. 2014;49(8):1016–21. 24777183 10.1038/bmt.2014.78

[7] Foroughi S, Wong HL, Gately L, Lee M, Simons K, Tie J, et al. Re-inventing the randomized controlled trial in medical oncology: the registry-based trial. Asia Pac J Clin Oncol. 2018;14(6):365–73. 29947051 10.1111/ajco.12992

[8] Human Research Ethics Committees (HRECs) review research proposals involving human participants to ensure that they are ethically acceptable: National Health and Medical Research Council; 2022. Available from: https://www.nhmrc.gov.au/research-policy/ethics/human-research-ethics-committees.

[9] Research governance and site specific assessment: Victorian Department of Health; 2024. Available from: https://www.clinicaltrialsandresearch.vic.gov.au/__data/assets/pdf_file/0020/171146/Research-Governance-SSA-Process-and-Practice.-March-2024.pdf.

[10] Purple Translational Registry: WEHI; 2024. Available from: https://purplepancreas.pixo.com.au/page/83/registry-data-projects.

[11] James S, Rao SV, Granger CB. Registry-based randomized clinical trials–a new clinical trial paradigm. Nat Rev Cardiol. 2015;12(5):312–6. 25781411 10.1038/nrcardio.2015.33

[12] Lauer MS, D’Agostino RB Sr. The randomized registry trial–the next disruptive technology in clinical research? N Engl J Med. 2013;369(17):1579–81. 23991657 10.1056/NEJMp1310102

[13] Baba A, Tay J, Sammy A, Douglas WA, Goren K, Krause KR, et al. Paper I: heterogeneous use of registry data for participant identification and primary outcome ascertainment is found in registry-based randomized controlled trials: a scoping review. J Clin Epidemiol. 2023;159:289–99. 37146658 10.1016/j.jclinepi.2023.04.016

[14] Ahern S, Gabbe BJ, Green S, Hodgson CL, Wood EM, Zalcberg Oam JR, et al. Realising the potential: leveraging clinical quality registries for real world clinical research. Med J Aust. 2022;216(6):273–7. 35267192 10.5694/mja2.51443

[15] Frobert O, Lagerqvist B, Olivecrona GK, Omerovic E, Gudnason T, Maeng M, et al. Thrombus aspiration during ST-segment elevation myocardial infarction. N Engl J Med. 2013;369(17):1587–97. 23991656 10.1056/NEJMoa1308789

[16] Buccheri S, Sarno G, Fröbert O, Gudnason T, Lagerqvist B, Lindholm D, et al. Assessing the nationwide impact of a registry-based randomized clinical trial on cardiovascular practice. Circ Cardiovasc Interv. 2019;12(3):e007381. 30841711 10.1161/CIRCINTERVENTIONS.118.007381

[17] Erlinge D, Koul S, Eriksson P, Scherstén F, Omerovic E, Linder R, et al. Bivalirudin versus heparin in non-ST and ST-segment elevation myocardial infarction-a registry-based randomized clinical trial in the SWEDEHEART registry (the VALIDATE-SWEDEHEART trial). Am Heart J. 2016;175:36–46. 27179722 10.1016/j.ahj.2016.02.007

[18] Krause KR, Tay J, Douglas WA, Sammy A, Baba A, Goren K, et al. Paper II: thematic framework analysis of registry-based randomized controlled trials provided insights for designing trial ready registries. J Clin Epidemiol. 2023;159:330–43. 37146660 10.1016/j.jclinepi.2023.04.015

[19] Yan MK, Adler NR, Heriot N, Shang C, Zalcberg JR, Evans S, et al. Opportunities and barriers for the use of Australian cancer registries as platforms for randomized clinical trials. Asia Pac J Clin Oncol. 2022;18(4):344–52. 34811922 10.1111/ajco.13670

[20] Li G, Sajobi TT, Menon BK, Korngut L, Lowerison M, James M, et al. Registry-based randomized controlled trials - what are the advantages, challenges, and areas for future research? J Clin Epidemiol. 2016;80:16–24. 27555082 10.1016/j.jclinepi.2016.08.003

[21] Prang KH, Karanatsios B, Zhang A, Verbunt E, Wong HL, Wong V, et al. “Nothing to lose and the possibility of gaining”: a qualitative study on the feasibility and acceptability of registry-based randomised controlled trials among cancer patients and clinicians. Trials. 2023;24(1):92. 36747274 10.1186/s13063-023-07109-2 PMC9902247

[22] Tong A, Sainsbury P, Craig J. Consolidated criteria for reporting qualitative research (COREQ): a 32-item checklist for interviews and focus groups. Int J Qual Health Care. 2007;19(6):349–57. 17872937 10.1093/intqhc/mzm042

[23] Nyimbili F, Nyimbili L. Types of purposive sampling techniques with their examples and application in qualitative research studies. British Journal of Multidisciplinary and Advanced Studies. 2024;5(1):90–9.

[24] Prang KH, Karanatsios B, Verbunt E, Wong HL, Yeung J, Kelaher M, Gibbs P. Clinical registries data quality attributes to support registry-based randomised controlled trials: a scoping review. Contemporary clinical trials. 2022;119:106843. 10.1016/j.cct.2022.106843 35792338

[25] Braun VCV. Using thematic analysis in psychology. Qual Res Psychol. 2006;3(2):77–101.

[26] Fereday J M-CE. Demonstrating rigor using thematic analysis: a hybrid approach of inductive and deductive coding and theme development. Int J Qual Methods. 2006;5(1):80–9.

[27] Kim SYH, Miller FG. Informed consent for pragmatic trials — the integrated consent model. N Engl J Med. 2014;370(8):769–72. 24552326 10.1056/NEJMhle1312508

[28] Anderson ML, Califf RM, Sugarman J. Ethical and regulatory issues of pragmatic cluster randomized trials in contemporary health systems. Clin Trials. 2015;12(3):276–86. 25733677 10.1177/1740774515571140 PMC4498459

[29] Goldstein CE, Weijer C, Brehaut JC, Fergusson DA, Grimshaw JM, Horn AR, Taljaard M. Ethical issues in pragmatic randomized controlled trials: a review of the recent literature identifies gaps in ethical argumentation. BMC medical ethics. 2018;19:1–0. 10.1186/s12910-018-0253-x PMC5827974 29482537

[30] Kalkman S, van Thiel GJMW, Zuidgeest MGP, Goetz I, Pfeiffer BM, Grobbee DE, et al. Series: pragmatic trials and real world evidence: paper 4. Informed consent. J Clin Epidemiol. 2017;89:181–7. 28502809 10.1016/j.jclinepi.2017.03.019

[31] Nicholls SG, Carroll K, Zwarenstein M, Brehaut JC, Weijer C, Hey SP, Goldstein CE, Graham ID, Grimshaw JM, McKenzie JE, Fergusson DA. The ethical challenges raised in the design and conduct of pragmatic trials: an interview study with key stakeholders. Trials. 2019;20:1–6. 10.1186/s13063-019-3899-x PMC6929346 31870433

[32] National Critical Research Infrastructure Initiative-2023 innovative trials: Australian Government-Medical Research Future Fund; 2023. Available from: https://www.grants.gov.au/Fo/Show?FoUuid=f54f2ea2-3a5d-48f0-84c9-8e23862f004a.

[33] RTI International. Registry trials project expert interview. USA: RTI International; 2016.

[34] National Clinical Trials Framework and user guide (NCTGF): Australian Commission on Safety and Quality in Health Care (ACSQHC). 2022. Available from: https://www.safetyandquality.gov.au/standards/national-clinical-trials-governance-framework.

[35] Registry based trials: Victorian Comprehensive Cancer Centre Alliance (VCCC); 2023. Available from: https://vcccalliance.org.au/research/clinical-trial-innovations/registry-trials/.

[36] Registry randomised trials workshop: Australian Clinical Trials Alliance (ACTA). 2020 Available from: https://clinicaltrialsalliance.org.au/?s=registry+trials.

[37] Newman AB, Avilés-Santa ML, Anderson G, Heiss G, Howard WJ, Krucoff M, et al. Embedding clinical interventions into observational studies. Contemp Clin Trials. 2016;46:100–5. 26611435 10.1016/j.cct.2015.11.017 PMC5626440

